# Gallic Acid Improves Muscular Function Through Enhanced Myoblast Myogenesis in Mice

**DOI:** 10.1002/fsn3.70667

**Published:** 2025-07-18

**Authors:** Sangsoo Lee, Dong‐Yup Han, Kee K. Kim

**Affiliations:** ^1^ Department of Biochemistry College of Natural Sciences, Chungnam National University Daejeon Republic of Korea; ^2^ Department of Sports and Leisure Studies Daegu University Gyeongsangbuk‐do Republic of Korea

**Keywords:** exercise performance, gallic acid, muscle function, myogenesis, natural supplement

## Abstract

Skeletal muscle health is crucial for maintaining physical function and metabolic homeostasis. This study investigated the effects of gallic acid, a naturally occurring phenolic compound, on myogenesis and muscle function. In vitro experiments using mouse primary myoblasts demonstrated that gallic acid (10 μg/mL) enhanced myogenic differentiation, evidenced by increased Myh3 protein expression (3.3‐fold under growth conditions and 1.3‐fold under differentiation conditions) and enhanced myotube formation in both conditions. In vivo studies were conducted using C57BL/6N mice fed either a control diet or a 0.2% gallic acid‐supplemented diet for 8 weeks. While gallic acid supplementation did not affect body weight, food intake, or general metabolic parameters, it significantly increased soleus muscle mass (10.56 ± 1.35 mg vs. 8.28 ± 0.68 mg in controls). Furthermore, gallic acid‐fed mice showed improved muscle function, with increased running distance (345.0 ± 28.8 m vs. 262.2 ± 58.6 m), extended time to exhaustion (24.2 ± 1.2 min vs. 20.1 ± 3.0 min), and enhanced grip strength (182.2 ± 19.4 N vs. 157.3 ± 11.5 N). These findings suggest that gallic acid could serve as a promising natural supplement for improving muscle function and exercise performance.

## Graphical abstract

Gallic acid treatment enhances myogenesis in primary myoblasts, with particularly strong effects under growth conditions. Dietary supplementation with gallic acid increases soleus muscle mass and improves exercise performance in mice. These findings suggest gallic acid promotes muscle synthesis and could serve as a natural supplement for improving muscle function.

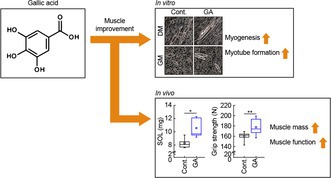

## 1 Introduction

Skeletal muscle, comprising approximately 40% of total body mass, contributes crucial roles in not only movement or strength but also metabolism and overall health (Butcher et al. 2018; Szaroszyk et al. 2022; Gehrig et al. 2010). Maintaining muscle function is essential for quality of life, particularly in the elderly. However, aging and various pathological conditions, such as sarcopenia and muscular dystrophies, can affect muscle function, leading to muscle weakness and dysfunction (Lauretani et al. 2003). Myogenesis, the process of muscle formation and regeneration, is fundamental for muscle development and maintenance (McCarthy et al. 2011; Joe et al. 2010). Myoblasts, muscle progenitor cells, differentiate into muscle fibers through myogenesis and form long, thick myotubes (Konigsberg 1963). Myogenesis is controlled by various molecular mechanisms, and the expression of myogenic proteins, as well as the length and thickness of the myotubes, are key indicators of myogenesis (Cuenda and Cohen 1999; Conejo et al. 2001).

Gallic acid (3,4,5‐trihydroxybenzoic acid), a naturally occurring phenolic compound, is widely distributed in various plants, fruits, and herbs (Pal singh et al. 2019; Jaijoy et al. 2010). Gallic acid has been extensively studied for its diverse biological activities, including antioxidant, anti‐inflammatory, and anticancer properties (Badavi et al. 2014; Kroes et al. 1992; Zhao and Hu 2013). Recent studies have demonstrated that gallic acid can influence various cellular processes, including cell proliferation, differentiation, and metabolism (Ferk et al. 2011; Pham et al. 2024). Additionally, gallic acid has demonstrated its ability to improve various pharmaceutical functions through its ability to modulate cellular signaling pathways (Hsieh et al. 2016). Despite the well‐known positive effects of gallic acid and recent studies revealing that phenolic compounds can affect muscle metabolism, the positive effects of gallic acid on muscle function and myogenesis have not been thoroughly investigated (Lee et al. 2017; Kuppusamy et al. 2019; Orzechowski et al. 2002). Understanding the beneficial effects of gallic acid could suggest valuable insights on a natural supplement for maintaining and improving muscle health. This study aimed to investigate the effects of gallic acid on myogenesis and muscle function. The effects of gallic acid treatment on primary myoblast differentiation and myotube formation were determined under various conditions. Furthermore, the effects of gallic acid supplementation on muscle mass and function in mice were also evaluated. These findings highlight the promising role of gallic acid in enhancing muscle health and function.

## 2 Materials and Methods

### 2.1 Isolation and Culture of Mouse Primary Myoblasts

Primary myoblasts were isolated from the hind limb skeletal muscles of 3‐day‐old mice. Muscle tissue was dissected, removing excess bone and fat, and digested in a 1:1 mixture of collagenase D (1.5 U/mL) and dispase (2.4 U/mL) for 1 h. The isolated myoblasts were collected by centrifugation (300 × *g*, 5 min) and resuspended in growth medium containing Ham's F‐10 medium supplemented with 10% calf serum (GIBCO, NY, USA), 1% penicillin–streptomycin, and 2.5 μg/mL fibroblast growth factor. Cells were cultured on poly‐L‐ornithine (PLO, Sigma, USA)‐coated dishes for maintenance or collagen‐coated dishes (Santa Cruz Biotechnology, CA, USA) for differentiation at 37°C in a humidified 5% CO_2_ atmosphere. For myogenic differentiation, when cells reached approximately 90% confluence, the medium was replaced with differentiation medium consisting of DMEM supplemented with 2% horse serum (Gibco, NY, USA) and 1% penicillin–streptomycin.

### 2.2 Immunoblot Analysis

Whole‐cell lysates were prepared from mouse primary myoblasts using ice‐cold Tris‐Triton buffer containing protease inhibitor cocktail (Roche Applied Science, Basel, Switzerland). Proteins were separated by sodium dodecyl sulfate‐polyacrylamide gel electrophoresis (SDS‐PAGE) and transferred to nitrocellulose membranes (Pall Life Science, Port Washington, NY, USA). The membranes were blocked with 5% skim milk in PBS containing 0.1% Tween 20 for 1 h at room temperature, followed by overnight incubation with primary antibodies against Myh3 (1:500, SC‐53091, Santa Cruz) and GAPDH (1:2000, SC‐47724, Santa Cruz). After washing with PBST, immunoreactive proteins were detected using horseradish peroxidase‐conjugated secondary antibodies (Abcam, USA) and visualized using the Super Signal system (Pierce Chemical, USA).

### 2.3 Animals and Experimental Diets

Twenty‐week‐old male C57BL/6N mice (Japan SLC Inc., Japan) were housed under controlled conditions (22°C ± 2°C, 50% ± 5% relative humidity, 12‐h light/dark cycle) with *ad libitum* access to food and water. Male mice were specifically selected for this study to minimize the potential influence of hormonal fluctuations on muscle metabolism and exercise performance, as estrogen has been shown to affect muscle satellite cell activation, protein synthesis, and antioxidant capacity. After 7 days of acclimatization, the mice were randomly divided into two groups (*n* = 5/group): control (normal diet) and gallic acid‐supplemented (0.2% gallic acid‐containing diet) groups. The experimental diets were administered for 8 weeks, with weekly monitoring of food intake and body weight. All animal procedures were approved by the Animal Experimental Ethics Committee of Chungnam National University (Daejeon, Korea) and conducted according to institutional guidelines.

### 2.4 Serum Biochemical Analysis

Serum biochemical parameters were analyzed using an autoanalyzer (AU680; Olympus, USA). The measured parameters included alanine transaminase (ALT), blood urea nitrogen (BUN), total cholesterol, high‐density lipoprotein (HDL), and low‐density lipoprotein (LDL) levels.

### 2.5 Treadmill Exercise Test and Grip Strength Test

Muscle endurance was evaluated after 5 weeks of gallic acid supplementation using a treadmill exercise test. Mice were subjected to a progressive running protocol starting at 10 cm/s for 3 min with 0% slope, followed by gradual speed increases to 15 cm/s and then to 50 cm/s until exhaustion. Exhaustion was defined as the inability to maintain running for 10 s. Grip strength was measured with all four limbs by a Grip Strength Test Meter (Bioseb, Chaville, France).

### 2.6 Statistical Analysis

Results are expressed as mean ± standard error of the mean (SEM). Statistical comparisons between groups were performed using two‐tailed Student's *t*‐test (SPSS software, version 12.0; BMI). Statistical significance was set at *p* < 0.05.

## 3 Results

### 3.1 Effects of Gallic Acid on Myogenesis of Mouse Primary Myoblasts

**FIGURE 1:**
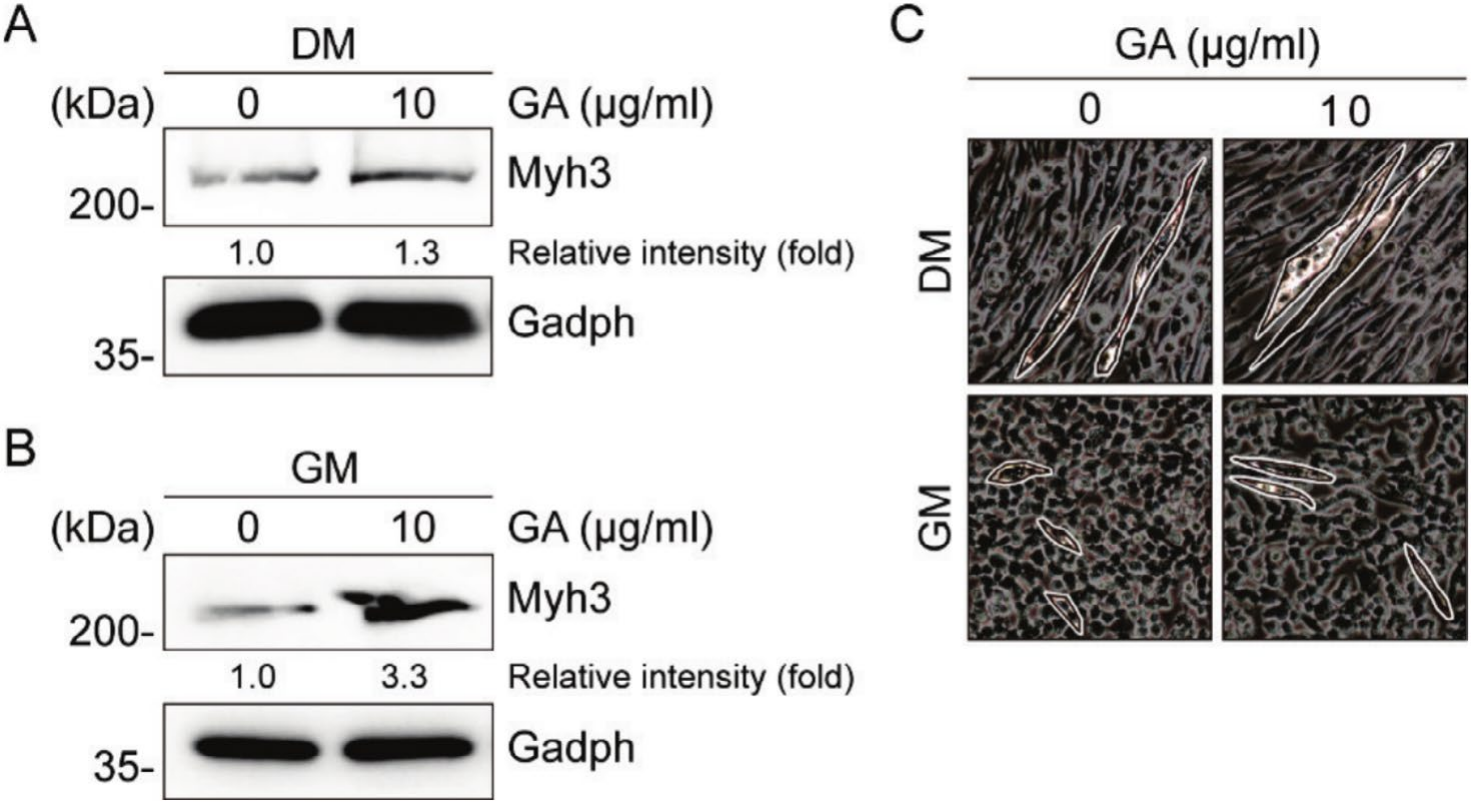
Gallic acid enhances myogenesis in mouse primary myoblasts (A). Immunoblot analysis of Myh3 expression in primary myoblasts treated with 10 μg/mL gallic acid under differentiation conditions for 3 days (B). Immunoblot analysis of Myh3 expression in primary myoblasts treated with 10 μg/mL gallic acid under growth conditions for 2 days. Gapdh served as loading control. Band intensities were quantified using ImageJ software. (C) Representative images of myotube formation under growth (GM) and differentiation (DM) conditions. Myotubes are outlined in each image.

The effects of gallic acid on myogenesis were evaluated by treating mouse primary myoblasts with 10 μg/mL gallic acid under both differentiation and growth conditions. Under differentiation conditions (3 days), gallic acid treatment increased Myh3 protein expression by 1.3‐fold compared to control (Figure 1A). More notably, under growth conditions (2 days), gallic acid treatment led to a 3.3‐fold increase in Myh3 protein expression (Figure 1B). Additionally, enhanced myotube formation was observed in both conditions following gallic acid treatment (Figure 1C). These results demonstrate that gallic acid promotes myogenesis in mouse primary myoblasts, with particularly strong effects under growth conditions.

### 3.2 Body Weight and Serum Metabolic Profiles in Gallic Acid‐Fed Mice

**FIGURE 2:**
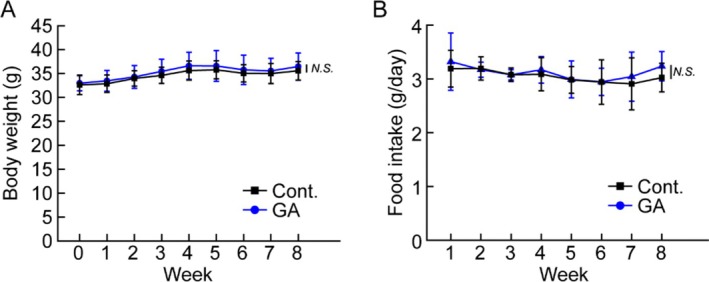
Effect of gallic acid supplementation on body weight and food intake: (A) Weekly body weight measurements of mice fed normal diet (Cont.) or 0.2% gallic acid‐supplemented diet (GA) for 8 weeks and (B) weekly food intake measurements of control and gallic acid‐supplemented groups. Data are presented as means ± SD (*n* = 5/group). N.S., not significant.

**FIGURE 3:**
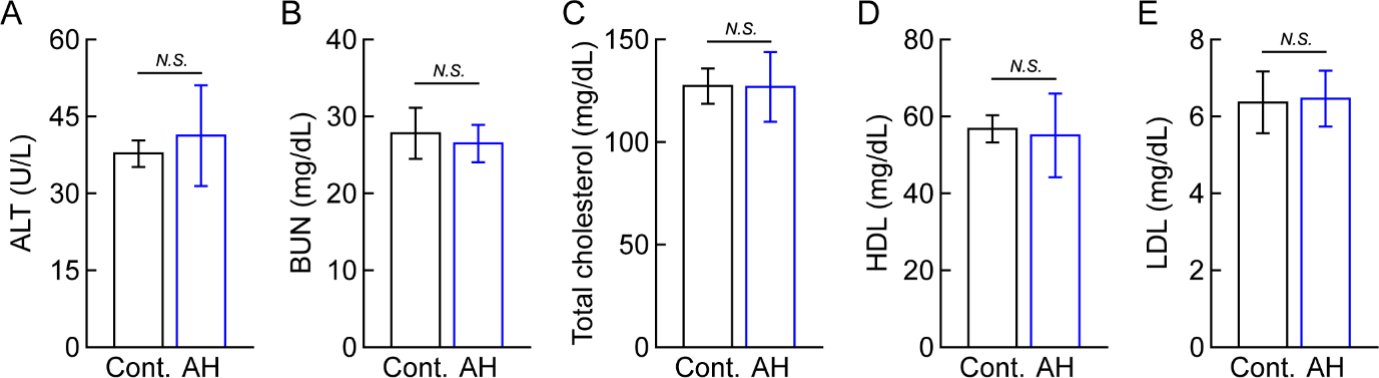
Blood biochemical parameters in control and gallic acid‐supplemented mice (A–E). Serum levels of (A) alanine transaminase (ALT), (B) blood urea nitrogen (BUN), (C) total cholesterol, (D) high‐density lipoprotein (HDL), and (E) low‐density lipoprotein (LDL) in mice fed normal diet (Cont.) or 0.2% gallic acid‐supplemented diet (GA) for 8 weeks. Data are presented as means ± SD (*n* = 5/group). N.S., not significant.

Following the observed enhancement of myogenesis in primary myoblasts, gallic acid was supplemented in a mouse model to assess its biological activity. To minimize the influence of growth‐related changes in body weight and muscle mass, 20‐week‐old mice were used for the in vivo experiments (Wang et al. 2020). Mice were fed with either a normal diet (control) or a diet containing 0.2% gallic acid (GA) for 8 weeks, allowing voluntary food consumption. Body weight monitoring showed no significant differences between groups, with final average weights of 35.6 ± 1.9 g and 36.5 ± 2.8 g in control and GA groups, respectively (Figure 2A). Similarly, food intake remained comparable between groups, with week 8 consumption averaging 3.02 ± 0.27 g in controls and 3.24 ± 0.27 g in GA‐fed mice (Figure 2B). Considering the gallic acid content in the diet, this indicated that GA‐fed mice ingested approximately 178 mpk of gallic acid daily over the 8‐week period. Serum analysis revealed no significant differences in ALT, BUN, total cholesterol, HDL, and LDL levels between groups (Figure 3), indicating that gallic acid supplementation did not alter overall metabolic parameters.

### 3.3 Improvement of Muscular Function in Gallic Acid‐Fed Mice

**FIGURE 4:**
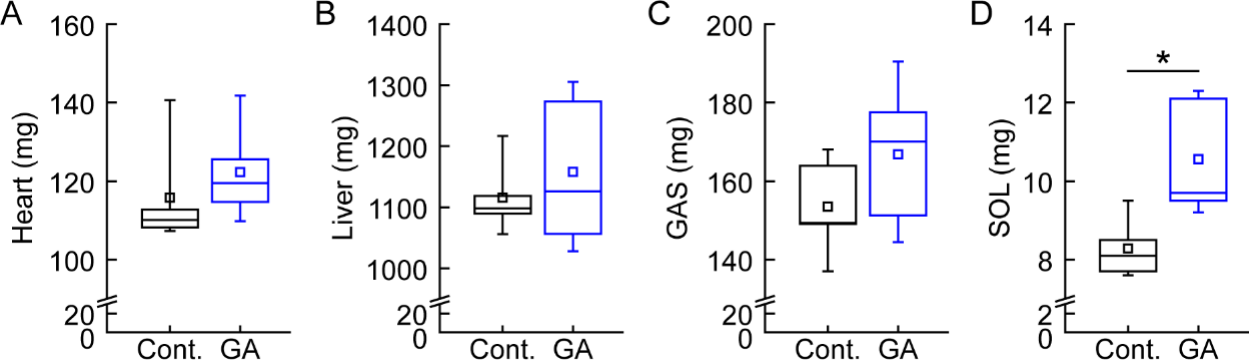
Effect of gallic acid supplementation on organ weights (A–D). Weight of (A) heart, (B) liver, (C) gastrocnemius muscle, and (D) soleus muscle in mice fed normal diet (Cont.) or 0.2% gallic acid‐supplemented diet (GA) for 8 weeks. Data are presented as means ± SD (*n = 5/group). N*.S., not significant; *p < 0.05 (paired *t*‐test).

In advance of evaluating muscle function, the effects of gallic acid supplementation on major organ weights were evaluated. While heart, liver, and gastrocnemius muscle weights showed no significant differences between groups (Figure 4A–C), the soleus muscle mass was significantly higher in GA‐fed mice (10.56 ± 1.35 mg) compared to controls (8.28 ± 0.68 mg; Figure 4D).

**FIGURE 5:**
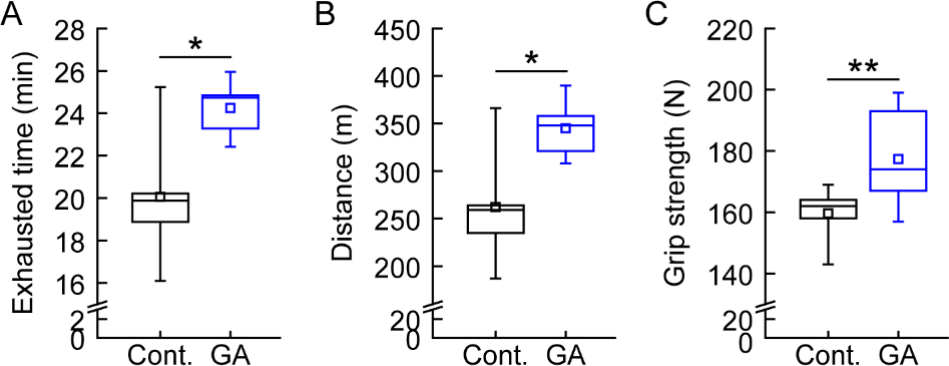
Gallic acid supplementation enhances muscular function (A, B) Treadmill endurance test results showing (A) running distance and (B) time to exhaustion in mice subjected to progressively increasing speeds. (C) Hindlimb grip strength measurements of mice fed normal diet (Cont.) or 0.2% gallic acid‐supplemented diet (GA) for 8 weeks. Data are presented as means ± SD (*n* = 5/group). *p < 0.05, **p < 0.01 (paired *t*‐test).

Muscle function was then evaluated through endurance and strength tests. In treadmill endurance tests, GA‐fed mice exhibited significantly longer distances (345.0 ± 28.8 m vs. 262.2 ± 58.6 m in controls; Figure 5A) and increased time to exhaustion (24.2 ± 1.2 min vs. 20.1 ± 3.0 min in controls; Figure 5B). Additionally, grip strength measurements revealed enhanced muscle strength in GA‐fed mice (182.2 ± 19.4 N vs. 157.3 ± 11.5 N in controls; Figure 5C). These results demonstrate that gallic acid supplementation improves muscle mass and muscle function in mice.

## 4 Discussion

This study demonstrates that gallic acid, a natural phenolic compound, enhances myogenesis in in vitro assays and improves muscle function in in vivo models. These findings provide novel insights into the applications of gallic acid as a natural supplement for maintaining and improving muscle health. The in vitro experiments revealed that gallic acid significantly enhanced myogenesis in primary myoblasts, as evidenced by increased Myh3 expression and enhanced myotube formation. Notably, the effect was more pronounced under growth conditions (3.3‐fold increase) compared to differentiation conditions (1.3‐fold increase), suggesting that gallic acid might primarily influence the early stages of myogenesis (Agarwal et al. 2020; Sharma et al. 2018). This finding aligns with previous studies showing that various phenolic compounds can modulate myogenic differentiation through different signaling pathways. As a previous study highlighted the antioxidant and anti‐inflammatory properties of gallic acid following 8‐week administration in animal models, the in vivo assay of this study was designed with mice fed a gallic acid‐containing diet for 8 weeks (Ramezani Ali Akbari et al. 2019). The in vivo study demonstrated that gallic acid supplementation led to significant improvements in muscle function without affecting body weight or general metabolic parameters. The increase in soleus muscle mass, but not in gastrocnemius muscle, suggests that gallic acid might have muscle type‐specific effects (Foster et al. 2012). The soleus muscle, predominantly composed of type I slow‐twitch fibers, is particularly important for endurance and postural maintenance (Lee et al. 2021; Bohm et al. 2021). This selective effect could explain the improved endurance performance observed in the treadmill tests.

The enhanced muscle function, demonstrated by increased running distance, time to exhaustion, and grip strength, suggests that gallic acid supplementation may have practical applications in improving physical performance and maintaining muscle health. These improvements occurred without changes in food intake or body weight, indicating that the effects of gallic acid were not due to altered energy balance or overall growth. The blood profile of gallic acid‐fed mice is supported by biochemical analyses, showing no significant changes in liver function (ALT), kidney function (BUN), or lipid profile parameters. This is consistent with previous studies reporting the safety of gallic acid consumption. However, longer‐term studies would be valuable to confirm the safety of chronic supplementation.

These findings may have particular relevance for conditions characterized by muscle weakness or dysfunction, such as sarcopenia and age‐related muscle wasting. While existing studies have highlighted gallic acid as a muscle relaxant and documented its protective effects on muscle tissue, this study uniquely demonstrates gallic acid's capacity to enhance myogenesis and improve muscle function (Yu et al. 2025). This suggests its potential as a natural supplement for maintaining muscle health during aging. Additionally, its presence in various foods and its established safety profile make it an attractive candidate for dietary supplementation. Several mechanisms might underlie the observed effects of gallic acid on muscle function (Asdaq et al. 2021; Jin et al. 2018). Previous studies have shown that phenolic compounds can modulate various signaling pathways involved in muscle development and function, including AMPK, PGC‐1α, and antioxidant responses (Tanaka et al. 2020; Zarei et al. 2023; Jrad et al. 2014). Related research has highlighted the beneficial effects of gallic acid on mitochondrial dynamics and myogenic gene expression in fully differentiated myotubes (Chang et al. 2021). Notably, the observation in this study that gallic acid improved the myogenesis of myoblasts in growth medium suggests a novel mechanism whereby gallic acid may enhance signaling pathways that trigger the transition of proliferative myoblasts into the differentiation stage. This represents a distinct functional aspect of gallic acid beyond its known antioxidant properties. Although mitochondrial function and antioxidant parameters were not directly assessed in the present study, these pathways may contribute to the observed effects, particularly during the later stages of myogenesis when significant mitochondrial adaptations occur. Future studies should investigate these specific molecular mechanisms through which gallic acid enhances myogenesis and improves muscle function, with particular attention to redox signaling pathways and mitochondrial biogenesis during the transition from myoblast proliferation to differentiation.

This study has some limitations that should be addressed in future research. First, given male mice were used in the present study, sex‐specific differences in response to gallic acid supplementation should be investigated. Second, dose–response studies would be valuable for determining optimal supplementation levels. Third, the long‐term effects of gallic acid supplementation remain to be established. Finally, human studies investigating the effects of gallic acid on adult muscle are needed to confirm the translational potential of these findings.

## 5 Conclusion

This study provides evidence that gallic acid enhances myogenesis and improves muscle function, particularly endurance and strength, without affecting overall metabolic parameters. These findings suggest that gallic acid could be a promising natural supplement for maintaining and improving muscle health. Future studies should focus on elucidating the molecular mechanisms involved and investigating the applications in various conditions affecting muscle function.

## Author Contributions

**Sangsoo Lee:** data curation (equal), formal analysis (equal), validation (equal), visualization (equal), writing – original draft (equal). **Dong‐Yup Han:** conceptualization (equal). **Kee K. Kim:** conceptualization (equal), supervision (lead), writing – review and editing (lead).

## Ethics Statement

Research and animal care protocols were approved by the Animal Experimental Ethics Committee of the Chungnam National University (Daejeon, Korea, approval no. 202410A‐CNU‐209) and were performed in accordance with the institutional guidelines.

## Conflicts of Interest

The authors declare no conflicts of interest.

## Supporting information


Data S1.

## Data Availability

The data that support the findings of this study are available from the corresponding author upon reasonable request.
