# Supplementary figures and images for: Gallic Acid Improves Muscular Function Through Enhanced Myoblast Myogenesis in Mice
Source: Food Sci Nutr. 2025 Jul 18;13(7):e70667. doi: 10.1002/fsn3.70667 (PMC12274160; doi:10.1002/fsn3.70667)

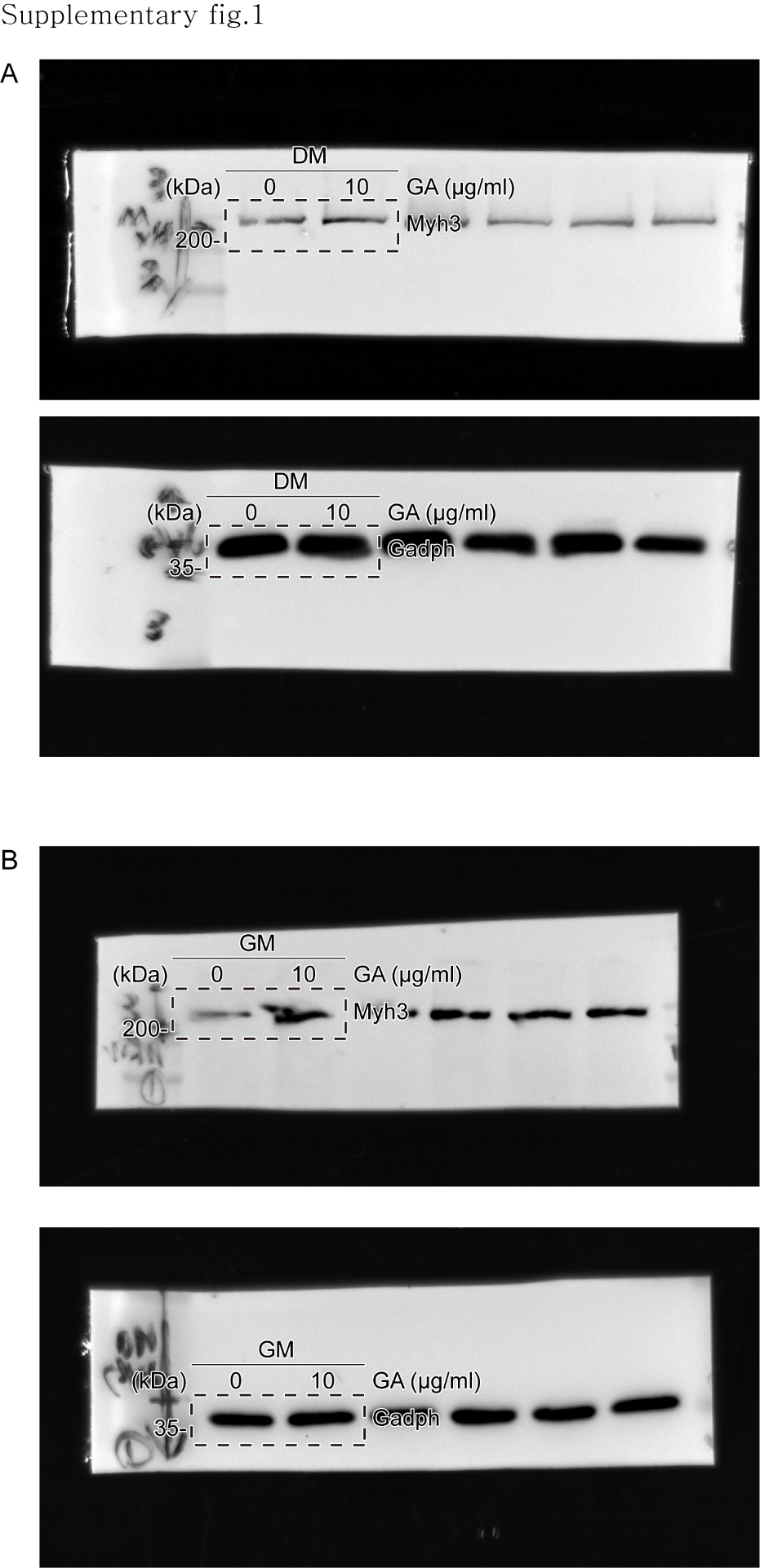

Supplement: Supplementary file 1 — Data S1. [file FSN3-13-e70667-s001.docx]
